# Supplementary material for: COVID-19-Related Social Isolation Predispose to Problematic Internet and Online Video Gaming Use in Italy
Source: Int J Environ Res Public Health. 2022 Jan 29;19(3):1539. doi: 10.3390/ijerph19031539 (PMC8835465; doi:10.3390/ijerph19031539)
Supplement: Supplementary file 1 [file ijerph-19-01539-s001.zip › ijerph-1522126-supplementary.pdf]

Table S1. Correlations I

	IAT Total	IAT Salience	IAT Excessive use	IAT Neglect Work	IAT Anticipa-tion	IAT Lack of Control	IAT Neglect Social Life	IGDS	BSMAS	DASS Total	DASS Depression	DASS Anxiety	DASS Stress	SAI	BWS
**IAT Total**	1	,816**	,825**	,736**	,608**	,020	,518**	,457**	,465**	-,346**	-,337**	-,278**	-,252**	-,018	-,027
IAT Salience	,816**	1	,618**	,504**	,444**	-,205**	,403**	,435**	,411**	-,361**	-,339**	-,317**	-,250**	-,015	-,023
IAT Excessive Use	,825**	,618**	1	,570**	,388**	-,195**	,342**	,428**	,436**	-,373**	-,355**	-,284**	-,294**	-,013	-,010
IAT Neglect Work	,736**	,504**	,570**	1	,352**	-,181**	,285**	,323**	,427**	-,337**	-,336**	-,237**	-,269**	-,066*	-,040
IAT Anticipa-tion	,608**	,444**	,388**	,352**	1	-,069**	,239**	,214**	,240**	-,201**	-,187**	-,184**	-,134**	-,001	-,010
IAT Lack Of Control	,020	-,205**	-,195**	-,181**	-,069**	1	-,139**	-,119**	-,166**	,300**	,289**	,215**	,245**	,043	,013
IAT Neglect So-cial Life	,518**	,403**	,342**	,285**	,239**	-,139**	1	,286**	,231**	-,155**	-,186**	-,092**	-,105**	,001	-,033
**IGDS**	,457**	,435**	,428**	,323**	,214**	-,119**	,286**	1	,203**	-,189**	-,202**	-,160**	-,110**	-,028	-,018
**BSMAS**	,465**	,411**	,436**	,427**	,240**	-,166**	,231**	,203**	1	-,367**	-,328**	-,283**	-,309**	-,106**	-,006
**DASS Total**	-,346**	-,361**	-,373**	-,337**	-,201**	,300**	-,155**	-,189**	-,367**	1	,863**	,797**	,851**	,031	-,032
DASS Depres-sion	-,337**	-,339**	-,355**	-,336**	-,187**	,289**	-,186**	-,202**	-,328**	,863**	1	,515**	,623**	,025	-,019
DASS Anxiety	-,278**	-,317**	-,284**	-,237**	-,184**	,215**	-,092**	-,160**	-,283**	,797**	,515**	1	,518**	,004	-,028
DASS Stress	-,252**	-,250**	-,294**	-,269**	-,134**	,245**	-,105**	-,110**	-,309**	,851**	,623**	,518**	1	,049	-,033
**SAI**	-,018	-,015	-,013	-,066*	-,001	,043	,001	-,028	-,106**	,031	,025	,004	,049	1	,013
**BWS**	-,027	-,023	-,010	-,040	-,010	,013	-,033	-,018	-,006	-,032	-,019	-,028	-,033	,013	1

**: Significant correlation at two-tailored p=0.01; *: Significant correlation at two-tailored p=0.05.

Table S2. Correlations II

	BWS	BIS Attent	BIS Motor	BIS Non planning	SAI	AQ Total	AQ Physical	AQ Verbal	AQ Anger	TAS Total	TAS DDF	TAS DIF	TAS EOT	IAT Total	IAT Salience	IAT Excessive Use	IAT Neglect Work	IAT Anticip	IAT Lack of control	IAT Neglect Social Life	IGDS	BSMAS
BWS	1	-,010	,013	-,016	,013	-,058*	,001	-,095**	-,047	-,055*	-,023	-,002	-,095**	-,027	-,023	-,010	-,040	-,010	,013	-,033	-,018	-,006
BIS Attent	-,010	1	,292**	-,043	-,044	-,301**	-,196**	-,147**	-,254**	,159**	,135**	,131**	,064*	,230**	,245**	,253**	,205**	,096**	-,191**	,144**	,185**	,194**
BIS Motor	,013	,292**	1	-,152**	-,092**	-,279**	-,203**	-,152**	-,258**	,066*	,040	,072**	,022	,099**	,091**	,125**	,101**	,061*	-,140**	,101**	,171**	,126**
BIS Non Planning	-,016	-,043	-,152**	1	,151**	,107**	,069*	,096**	,059*	,037	,019	,035	,022	-,046	-,052	-,059*	-,060*	,009	,092**	-,085**	-,090**	-,025
SAI	,013	-,044	-,092**	,151**	1	,189**	,095**	,126**	,138**	,106**	,065*	,055*	,105**	-,018	-,015	-,013	-,066*	-,001	,043	,001	-,028	-,106**
AQ Total	-,058*	-,301**	-,279**	,107**	,189**	1	,682**	,613**	,777**	-,034	-,095**	-,066*	,099**	-,217**	-,331**	-,307**	-,296**	-,143**	,629**	-,221**	-,202**	-,340**
AQ Physical	,001	-,196**	-,203**	,069*	,095**	,682**	1	,234**	,445**	-,049	-,043	-,051	-,005	-,092**	-,231**	-,203**	-,168**	-,095**	,642**	-,201**	-,156**	-,171**
AQ Verbal	-,095**	-,147**	-,152**	,096**	,126**	,613**	,234**	1	,360**	,084**	,017	,036	,129**	-,137**	-,141**	-,154**	-,191**	-,078**	,230**	-,124**	-,104**	-,175**
AQ Anger	-,047	-,254**	-,258**	,059*	,138**	,777**	,445**	,360**	1	-,049	-,061*	-,095**	,063*	-,121**	-,212**	-,177**	-,159**	-,060*	,388**	-,144**	-,120**	-,240**
TAS Total	-,055*	,159**	,066*	,037	,106**	-,034	-,049	,084**	-,049	1	,730**	,736**	,622**	,164**	,169**	,148**	,133**	,074**	-,029	,035	,083**	,080**
TAS DDF	-,023	,135**	,040	,019	,065*	-,095**	-,043	,017	-,061*	,730**	1	,332**	,205**	,151**	,158**	,135**	,154**	,074**	-,080**	,052	,073**	,118**
TAS DIF	-,002	,131**	,072**	,035	,055*	-,066*	-,051	,036	-,095**	,736**	,332**	1	,147**	,128**	,133**	,131**	,086**	,065*	-,027	,023	,008	,092**
TAS EOT	-,095**	,064*	,022	,022	,105**	,099**	-,005	,129**	,063*	,622**	,205**	,147**	1	,061*	,060*	,039	,038	,014	,049	-,001	,101**	-,050
IAT Total	-,027	,230**	,099**	-,046	-,018	-,217**	-,092**	-,137**	-,121**	,164**	,151**	,128**	,061*	1	,816**	,825**	,736**	,608**	,020	,518**	,457**	,465**
IAT Salience	-,023	,245**	,091**	-,052	-,015	-,331**	-,231**	-,141**	-,212**	,169**	,158**	,133**	,060*	,816**	1	,618**	,504**	,444**	-,205**	,403**	,435**	,411**
IAT Excessive Use	-,010	,253**	,125**	-,059*	-,013	-,307**	-,203**	-,154**	-,177**	,148**	,135**	,131**	,039	,825**	,618**	1	,570**	,388**	-,195**	,342**	,428**	,436**

IAT Neglect Work	-,040	,205**	,101**	-,060*	-,066*	-,296**	-,168**	-,191**	-,159**	,133**	,154**	,086**	,038	,736**	,504**	,570**	1	,352**	-,181**	,285**	,323**	,427**
IAT Antici-pation	-,010	,096**	,061*	,009	-,001	-,143**	-,095**	-,078**	-,060*	,074**	,074**	,065*	,014	,608**	,444**	,388**	,352**	1	-,069**	,239**	,214**	,240**
IAT Lack Of Control	,013	-,191**	-,140**	,092**	,043	,629**	,642**	,230**	,388**	-,029	-,080**	-,027	,049	,020	-,205**	-,195**	-,181**	-,069**	1	-,139**	-,119**	-,166**
IAT Neglect Social Life	-,033	,144**	,101**	-,085**	,001	-,221**	-,201**	-,124**	-,144**	,035	,052	,023	-,001	,518**	,403**	,342**	,285**	,239**	-,139**	1	,286**	,231**
IGDS	-,018	,185**	,171**	-,090**	-,028	-,202**	-,156**	-,104**	-,120**	,083**	,073**	,008	,101**	,457**	,435**	,428**	,323**	,214**	-,119**	,286**	1	,203**
BSMAS	-,006	,194**	,126**	-,025	-,106**	-,340**	-,171**	-,175**	-,240**	,080**	,118**	,092**	-,050	,465**	,411**	,436**	,427**	,240**	-,166**	,231**	,203**	1

**: Significant correlation at two-tailored p=0.01; *: Significant correlation at two-tailored p=0.05.
